# Licochalcone A suppresses pancreatic ductal adenocarcinoma progression by targeting eEF2K-mediated pyroptosis

**DOI:** 10.3389/fphar.2025.1595686

**Published:** 2025-06-11

**Authors:** Junjie Peng, Hiutung Chan, Wenqing Chen, Ken Kin-Lam Yung, King-Ho Cheung, Zhu Zhang

**Affiliations:** ^1^ Teaching and Research Division, School of Chinese Medicine, Hong Kong Baptist University, Kowloon, Hong Kong SAR, China; ^2^ Department of Science and Environmental Studies, The Education University of Hong Kong, Tai Po, Hong Kong SAR, China

**Keywords:** pancreatic ductal adenocarcinoma, licochalcone A, pyroptosis, autophagy, eEF2K

## Abstract

Pancreatic ductal adenocarcinoma (PDAC), a highly aggressive malignancy with increasing incidence and low survival rates, urgently requires novel therapeutic strategies to overcome the limitations posed by current treatment agents like gemcitabine. Licochalcone A (LHA), a bioactive flavonoid derived from *Glycyrrhiza* species, exhibits anticancer properties in multiple cancers, yet its efficacy and mechanisms in PDAC remain unexplored. This study aims to investigate the anticancer potential of LHA in PDAC. *In vitro* assays demonstrated that LHA dose-dependently inhibited PDAC cell proliferation and induced pyroptosis, a lytic inflammatory cell death, while autophagy inhibition synergistically enhanced the cytotoxicity of LHA. Furthermore, LHA suppressed the migration of PDAC cells. Mechanistically, molecular docking and functional studies revealed that LHA directly binds to eukaryotic elongation factor 2 kinase (eEF2K), reducing its expression and downstream phosphorylation of eukaryotic elongation factor 2 (p-eEF2). Notably, eEF2K overexpression reversed LHA-induced pyroptosis in PDAC cells. *In vivo*, LHA significantly reduced tumor growth and altered tumor histopathology in a PDAC xenograft model, along with downregulated eEF2K and upregulated pyroptosis executors (GSDMD/GSDME). Collectively, these findings identify LHA as a dual-function agent: a natural eEF2K inhibitor and a pyroptosis inducer with potent antitumor activity against PDAC. This study provides a foundational rationale for further clinical exploration of LHA as a promising chemotherapeutic agent or adjuvant to enhance PDAC treatment outcomes.

## Graphical Abstract

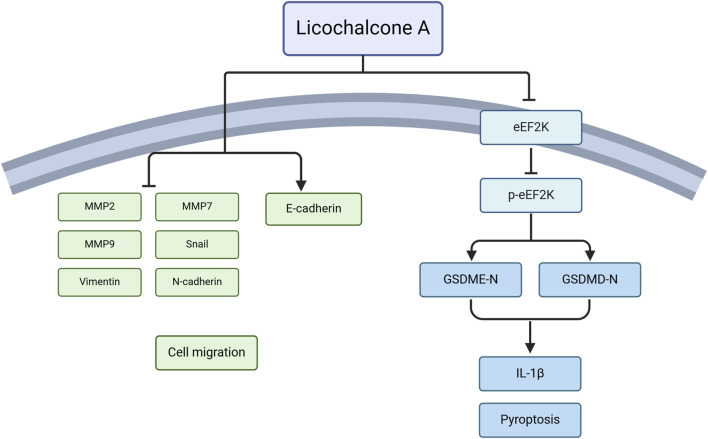

## 1 Introduction

Pancreatic ductal adenocarcinoma (PDAC) is the dominant type of pancreatic cancer, and its incidence rate has significantly increased recently (Grossberg et al., 2020; Mukund et al., 2024). The aggressive nature of PDAC—characterized by late diagnosis, rapid metastasis, and profound resistance to conventional therapies—underscores the urgent need for innovative treatment strategies (Dominguez et al., 2024). Gemcitabine, the first-line chemotherapy for PDAC, offers limited survival benefits due to intrinsic and acquired drug resistance, dose-limiting toxicity, and severe side effects such as myelosuppression and nephrotoxicity (Quiñonero et al., 2019; Zhang et al., 2022). Although combination regimens like FOLFIRINOX or gemcitabine–nab-paclitaxel have demonstrated improved outcomes, their clinical utility remains constrained by toxicity and marginal efficacy in advanced stages of PDAC (Principe et al., 2021; Klein-Brill et al., 2022). This therapeutic challenge stems from the predominant reliance of most antitumor agents on apoptosis-mediated tumor suppression, which is frequently subverted by the anti-apoptotic properties of tumor cells—a phenomenon potentially linked to their immunogenic inertness. Consequently, exploring non-apoptotic programmed cell death mechanisms holds significant promise for advancing cancer therapeutics. Pyroptosis, a recently identified pro-inflammatory programmed cell death modality, has demonstrated potent tumoricidal effects. Emerging evidence highlights the critical role of pyroptosis in PDAC pathogenesis, progression, and therapeutic response, underscoring its potential as a novel research frontier in PDAC treatment (Ding et al., 2023; Li J. et al., 2023).

Pyroptosis, distinct from apoptosis, triggers plasma membrane rupture via gasdermin pore formation, thus causing a release of proinflammatory cytokines (e.g., IL-1β and IL-18) that remodel the tumor microenvironment and enhance antitumor immunity (Meybodi et al., 2024; Liu et al., 2022; Imre, 2024). Cell pyroptosis has a “double-edged sword” effect in anti-tumor treatment (Liang et al., 2024; Tan et al., 2021). On the one hand, the release of inflammatory factors mediated by gasdermin protein activates immune responses, such as recruiting T cells and enhancing antigen presentation, directly killing tumors or synergizing with radiotherapy and chemotherapy (Li M. et al., 2023); on the other hand, excessive or persistent inflammation may promote angiogenesis, the formation of an immunosuppressive microenvironment, and even accelerate tumor metastasis (Ju et al., 2023). Its ultimate effect depends on the induction intensity, tumor type, and microenvironment status (Tu et al., 2023; Wang Y. et al., 2024). Recent studies suggested eukaryotic elongation factor 2 kinase (eEF2K) may undergo a crosstalk with pyroptotic pathways (Yu P. et al., 2019). eEF2K regulates protein synthesis by phosphorylating and inactivating eukaryotic elongation factor 2 (eEF2) and is a pivotal molecular target in PDAC (Karakas and Ozpolat, 2020; Rahman et al., 2022). eEF2K is overexpressed in cancers and correlates with poor prognosis as it promotes tumor survival under metabolic stress, enhances chemoresistance, and facilitates metastatic progression (Wang H. et al., 2024). The inhibition of eEF2K can sensitize cancer cells to apoptosis and suppress tumor growth, positioning it as a promising therapeutic target (Zhang et al., 2021).

However, existing synthetic eEF2K inhibitors face challenges such as off-target effects and poor pharmacokinetic properties, necessitating the discovery of natural compounds with improved specificity and safety. Natural products, particularly phytochemicals derived from traditional medicinal plants, have re-emerged as promising candidates for anticancer drug discovery due to their multitarget mechanisms, low-toxicity profiles, and historical validation in ethnopharmacology (Chunarkar-Patil et al., 2024). Licochalcone A (LHA), a bioactive flavonoid isolated from the roots and stolons of *Glycyrrhiza* species (licorice plants), has garnered attention for its broad-spectrum pharmacological properties, including anti-inflammatory, antioxidant, and anticancer activities (Zhu et al., 2023; Li et al., 2022). Preclinical studies highlight the ability of LHA to induce apoptosis, suppress angiogenesis, and inhibit metastasis in breast, liver, and lung carcinomas by modulating key pathways like PI3K/AKT and mTOR signaling (Hao et al., 2019). Although the apoptotic effects of LHA have been established in other cancers, this study uniquely investigates its capacity to trigger pyroptosis in PDAC, potentially leveraging this immunogenic death pathway to overcome chemoresistance in PDAC.

In this study, we investigated the antitumor effects of LHA on PDAC both *in vitro* and *in vivo* and elucidated the mechanism of these effects through focusing on eEF2K-regulated pyroptosis. These findings establish the preclinical potential of LHA as a chemotherapeutic agent or adjuvant for PDAC treatment.

## 2 Materials and methods

### 2.1 Materials

Phosphate-buffered saline (PBS), penicillin–streptomycin–neomycin (PSN) antibiotic mixture, Roswell Park Memorial Institute (RPMI) 1640 medium, Dulbecco’s modified Eagle medium (DMEM), trypsin–EDTA (0.25%), and Pierce™ BCA Protein Assay Kits were purchased from Thermo Fisher Scientific (Waltham, MA). Dimethyl sulfoxide (DMSO) and bovine serum albumin (BSA) were bought from Sigma-Aldrich, St. Louis, MO. The 3-(4,5-dimethylthiazol-2-yl)-2,5-diphenyl-tetrazolium bromide (MTT) assay kit was obtained from AMRESCO (Solon, OH, United States). The Cell‐Light™ EdU Apollo^®^567 *In Vitro* Imaging Kit was bought from Ribo Biological Co., Ltd. (Guangzhou, China). The reverse transcription kit for qPCR was bought from Takara (Otsu, Shiga, Japan). FxCycle™ PI/RNase staining solution, TRIzol^®^ Reagent, and the CyQUANT LDH Cytotoxicity Assay Kit were obtained from Invitrogen (Carlsbad, CA, United States). qPCR/real-time PCR reagents and the protein ladders in Western immunoblotting were bought from Bio-Rad Laboratories (Philadelphia, PA, United States). In Table 1, the primary antibodies used in Western immunoblotting are recorded. The secondary antibodies, goat anti-mouse IgG, and goat anti-rabbit IgG were obtained from Santa Cruz Biotechnology (Santa Cruz, CA, United States).

**TABLE 1:** Primary antibodies used in Western immunoblotting.

Antibody	Dilution	Manufacturer
eEF2K	1:1,000	CST
p-eEF2	1:1,000	CST
eEF2	1:1,000	CST
p62	1:1,000	CST
LC3	1:2,000	CST
GSDMD	1:500	CST
GSDME N-terminal	1:500	CST
IL-1β	1:3,000	Abcam
GAPDH	1:3,000	Santa Cruz

### 2.2 Reagents

Licochalcone A (CAS. no.: 58749-22-7; ≥98% purity) was obtained from Chengdu Must Bio-technology Co., Ltd. Gemcitabine (CAS. No: HY-17026; 99.72% purity) was bought from MedChemExpress. Chloroquine disphosphate salt (CAS no.: C6628-25 g; 98.5%–101.0% (EP)) was purchased from Sigma-Aldrich, St. Louis, MO.

### 2.3 Culture of PDAC cell lines

Human PDAC cell lines, MIA PaCa-2 and BxPc3, were obtained from Procell Life Science&Technology Co., Ltd. MIA PaCa-2 cells were cultured in DMEM with 10% FBS and 1% PSN. BxPc3 cells were cultured with 10% FBS plus 1% PSN in RPMI-1640 medium. All cells were incubated in an incubator at 37^o^C with 5% CO_2_ atmosphere. Once the cells reached approximately 80% confluency, they were passaged using trypsin–EDTA (0.25%).

### 2.4 MTT cell viability assay

The growth-inhibitory effect of PDAC and glioblastoma cancer cells was tested by using the MTT assay. The stock powder of LHA was dissolved in 0.01% DMSO. MIA PaCa-2 (9 × 10^3^/well) and BxPc3 (9 × 10^3^/well) cells were seeded in 96-well plates and adhered overnight. Cancer cells were treated with LHA (1, 2.5, 5, 7.5, 10, 12.5, and 15 µM) for 24, 48, and 72 h. Cells were incubated in the MTT solution (5 mg/mL) for an additional 1 h at 37^o^C once they reached the time points. After incubation with the MTT solution, the resulting formazan product was dissolved in DMSO; spectrophotometric analysis was performed at a 570 nm absorbance wavelength with a 650 nm reference wavelength.

### 2.5 Tumor colony formation assay

PDAC cancer cells were seeded in a six-well plate (0.7 × 10^3^ cells/well) and treated with LHA (7.5, 10, and 12.5 µM) for 24 h. After the drug incubation, the drug was replaced with DMEM with 10% FBS and 1% PSN and with RPMI-1640 with 10% FBS and 1% PSN, according to different cell lines. The cell colonies were washed with PBS after 12 days of cell culture. The fixation and staining process used 4% paraformaldehyde (PFA) and 0.3% crystal violet. ImageJ software was used for counting the number of colonies.

### 2.6 5-Ethynyl-2′-deoxyuridine (EdU) proliferation assay

MIA PaCa-2 (2.5 × 10^4^/well) and BxPc3 (3 × 10^4^/well) cells were seeded in four-well plates and allowed to adhere overnight. Cells were then treated with LHA (7.5, 10, and 12.5 µM) for 24 h. The EdU solution was used in the dark for staining the cells, following the manufacturer’s protocol (Ribo Biological Co., Ltd., Guangzhou, China). Then, the nuclei of cancer cells were stained with Hoechst 33324. Three random pictures were captured in each well using a compound fluorescent microscope (Nikon, Japan), and all the pictures were analyzed using ImageJ software.

### 2.7 Lactate dehydrogenase (LDH) cytotoxicity assay

PDAC cells were seeded in 96-well plates and treated with LHA for 24 h and 48 h. Following the drug treatment, 50 µL of the medium was collected from each well. After that, the LDH release level was measured using the CyQUANT LDH Cytotoxicity Assay Kit, following the manufacturer’s instructions (Invitrogen, Carlsbad, CA, United States); spectrophotometric analysis was performed at 490 nm absorbance wavelength with 680 nm reference wavelength.

### 2.8 Real-time polymerase chain reaction (RT-PCR) analysis

The cells were lysed using TRIzol reagent, and DNase 1 was used for total cellular RNA extraction. The single-strand cDNA was synthesized by using the reverse transcription kit (Otsu, Shiga, Japan). The SYBR green reaction mixture was applied in an ABI 7500 rapid real-time PCR system (Applied Biosystem, Waltham, MA) to perform real-time PCR. The β-actin endogenous control was used for normalizing the expression of gene data. The relative levels of gene expression were quantified using the formula 2-ΔCt, where ΔCt stands for the variation in the threshold cycle values between β-actin and gene targets. All the primer sequences are listed in Table 2 and were synthesized by Bio-Dream Technology Co., Ltd. (Shenzhen, Guangdong, China) and Life Technologies Co., Ltd. (Kwai Chung, New Territories, Hong Kong).

**TABLE 2:** Primers used for RT-PCR.

Gene	Primer sequence	
MMP2 Human	Forward	GGC​ACC​CAT​TTA​CAC​CTA​CA
	Reverse	CCA​AGG​TCA​ATG​TCA​GGA​GAG
MMP7 Human	Forward	GCT​CAC​TTC​GAT​GAG​GAT​GAA
	Reverse	AGG​AAT​GTC​CCA​TAC​CCA​AAG
MMP9 Human	Forward	ACA​AGC​TCT​TCG​GCT​TCT​G
	Reverse	GGT​ACA​GGT​CGA​GTA​CTC​CTT​A
Snail Human	Forward	CAG​ATG​AGG​ACA​GTG​GGA​AAG
	Reverse	GAG​ACT​GAA​GTA​GAG​GAG​AAG​GA
Vimentin Human	Forward	CAG​GAA​CAG​CAT​GTC​CAA​ATC
	Reverse	GGC​AGC​CAC​ACT​TTC​ATA​TTG
E-cadherin Human	Forward	CTC​GAC​ACC​CGA​TTC​AAA​GT
	Reverse	CCA​GGC​GTA​GAC​CAA​GAA​AT
N-cadherin Human	Forward	GAC​AGT​TCC​TGA​GGG​ATC​AAA​G
	Reverse	CGA​TTC​TGT​ACC​TCA​ACA​TCC​C
*β-actin* Human	Forward	TGG​CAC​CCA​GCA​CAA​TGA​A
	Reverse	CTA​AGT​CAT​AGT​CCG​CCT​AGA​AGC​A

### 2.9 Western blotting

MIA PaCa-2 (10 × 10^5^/well) and BxPc3 (9.5 × 10^5^/well) cells were seeded in a 60-mm dish and allowed to adhere overnight. Afterward, cells were treated with LHA (7.5, 10, and 12.5 µM). Once it reached the time point, they were harvested using cold PBS with 137 mM NaCl, 2.68 mM KCl, and 1.47 mM KHPO_4_. The cells were centrifuged in 2000 rcf for 5 min at 4^o^C and washed two times with cold PBS during the sample harvesting. After washing, the cell pellets were preserved and stored at −80^o^C. The RIPA buffer, including 50 mM Tris, 150 mM NaCl, 0.5% deoxycholate, 0.1% SDS, 2 mM EDTA, 0.1% Triton X-100, 10% glycerol, 1 mM phenylmethylsulfonyl fluoride, and 10 μg/mL aprotinin, was used for cell lysis. Then, the cells were centrifuged at 14,000 rcf for 20 min at 4^o^C, and the protein concentration was quantified using the Pierce™ BCA Protein Assay Kits. The total cellular protein was separated using 8%–15% SDS polyacrylamide gel. After the electrophoresis, the gel was transferred to a PVDF membrane (0.45 µM pore size). The membrane was blocked with 5% non-fatty milk for 1.5 h and then incubated with the target primary antibodies overnight at 4^o^C. On another day, the membrane was washed thrice with TBST before incubating with the secondary antibody at room temperature for 1 h. After secondary antibody incubation, the membrane was washed thrice again with TBST. Film development was performed using the chemiluminescence sensitivity matching X-ray film (Bio-Rad, United States). The housekeeping protein GAPDH was used as a standard for normalizing the relative density of each protein, which was quantified using Image J software.

### 2.10 Lentivirus overexpression of eEF2K

Lentivirus vectors carrying the overexpressed human eEF2K gene and green fluorescent protein (GFP) were designed and produced as “LV-EEF2K (100354-1).” The construct was bought from the Genchem Company (Shanghai, China). The virus was transfected into MIA PaCa-2 and BxPc3 cells at a multiplicity of infection of 10 using HitransGP for 16 h. After 16 h, the medium was replaced with fresh medium containing 10% FBS. Compound fluorescence microscopy (Nikon, Japan) and Western immunoblotting were used to confirm the overexpression of eEF2K in the transfected cells.

### 2.11 Light microscopy imaging

MIA PaCa-2 (50 × 10^4^/well) and BxPc3 (50 × 10^4^/well) cells were seeded in a six-well plate and allowed to adhere overnight. After the drug treatment, the cell morphology was visualized using a compound fluorescence microscope (Nikon, Japan) under the bright-field mode at 48 h. Four random pictures were captured from each well.

### 2.12 Scanning electron microscopy (SEM) imaging

MIA PaCa-2 and BxPc3 cells were seeded in a six-well plate and allowed to adhere overnight. Cells were then treated with LHA (12.5 µM). After 48 h of drug treatment, the cells were fixed with 2.5% glutaraldehyde overnight at 20°C. Cells were treated for 5 minutes at each concentration in a graded series of ethanol (30, 50, 70, 95, and 100%). Isoamyl acetate was progressively added to the sample as a substitute for alcohol, followed by critical-point drying. The dried samples were placed in a freezing drier overnight after being cooled to −80°C. Every sample was coated with gold–palladium and subjected to SEM imaging (S-3400 N; Hitachi Science Systems Ltd.).

### 2.13 Wound-healing cell migration assay

MIA PaCa-2 (80 × 10^4^/well) and BxPc3 (85 × 10^4^/well) cells were seeded in a six-well plate and allowed to adhere overnight. Once the cells reached 90% confluence, a wound was created by using a sterile pipette tip to draw a straight and thick scratch. The drug was dissolved in the medium with 2% FBS and then added to the cells. The results were visualized using a compound fluorescent microscope (Nikon, Japan) under a bright-field mode at 0 h and 24 h. ImageJ software was used to measure the distance of wound margins.

### 2.14 Subcutaneous tumor xenograft model of PDAC cells

All animal experimental protocols were carried out with approval from the Committee on Use of Human and Animal Subjects in Teaching and Research of Hong Kong Baptist University (REC/22-23/0446) and conducted under the Regulations of the Department of Health, Hong Kong SAR. Five-week-old male SPF-grade Balb/c-nude mice were obtained from Beijing Vital River Laboratory Animal Technology Co., Ltd. MIA PaCa-2 cells were cultured in DMEM with 10% FBS and 1% PSN. Cells were digested with trypsin until a suitable concentration for subcutaneous tumor injection in mice was reached. Experimental mice were inoculated in the right armpit (forelimb) with 0.2 mL of a cell suspension containing 5 × 10^7^ cells/mL. Tumor growth was observed regularly. When the tumor grew to an average volume of 100 mm^3^, mice were randomly divided into four groups (n = 7, valid data from at least six mice per group were used for analysis), namely, the control group, the positive control gemcitabine group 15 mg/kg, the low-dose LHA treatment group 25 mg/kg, and the high-dose LHA treatment group 50 mg/kg. Treatment mice received intraperitoneal injection every 2 days, except for the positive control gemcitabine group. Mouse body weight and tumor size were measured twice weekly. The tumor volume (mm^3^) was calculated using the formula = 1/2 × (tumor long diameter × tumor short diameter) (Mukund et al., 2024). After 21 days, tumors were excised and collected. The removed tumor specimens were embedded in paraffin and preserved in 10% formalin for histological analysis. The tumor tissues were cryopreserved and sectioned for hematoxylin and eosin (H&E) staining and immunofluorescence (IF) assessment.

### 2.15 Histopathological analysis of animal tissues

After removal, the tumor samples were embedded and preserved. Tumor samples were cut cryogenically (5 µM) and stained with (H&E; Sigma-50 Aldrich, St. Louis, MO). A magnification of ×63 was used for H&E imaging, and representative regions were examined using a compound fluorescent microscope (Nikon, Japan) under a bright-field mode.

### 2.16 Immunofluorescence staining

Tumor samples were excised, embedded, and kept. The samples were cryogenically sliced (5 µM) and incubated with primary antibodies, including eEF2K (CST; 1:200 dilution), GSDMD (CST; 1:200 dilution), and GSDME (CST; 1:200 dilution), overnight at 4°C. After the incubation, samples were washed with PBS, followed by associated secondary antibody incubation and DAPI staining in the dark at room temperature for 1 h and 5 min, respectively. Afterward, the tumor samples were visualized under the confocal microscope (Carl Zeiss, Oberkochen, Germany).

### 2.17 Molecular docking

The corresponding crystal structure of eEF-2K protein was obtained from the RCSB PDB database (PDB ID: 7SHQ). The Protein Preparation Wizard from Schrödinger software was then used to perform protein preprocessing and regenerate states of native ligands, H-bond assignment optimization, etc. LigPrep was used to preprocess the LHA ligand and generate all its 3D chiral conformations. Furthermore, SiteMap is used to predict the best binding site. Receptor Grid Generation sets the most proper enclosing box to wrap the predicted binding site. On this basis, the active site of the protein is obtained. Then, the processed ligand compound LHA was molecularly docked with the protein active site (using the highest precision XP docking). XP docking results refer to XP Gscore. The MM-GBSA analysis results refer to MM-GBSA dG Bind.

### 2.18 Statistical analysis

Data were presented as mean ± standard deviation (S.D.) of three replications and analyzed with a two-tailed Student’s t-test between two groups. One-way variance analysis (ANOVA), followed by post hoc Dunnett’s multiple comparisons test, was used for comparison of more than two groups. All data were presented using GraphPad Prism version 10.0 software (GraphPad, San Diego, CA, United States). *P* values <0.05 were considered statistically significant.

## 3 Results

### 3.1 LHA inhibited PDAC cell growth

**FIGURE 1:**
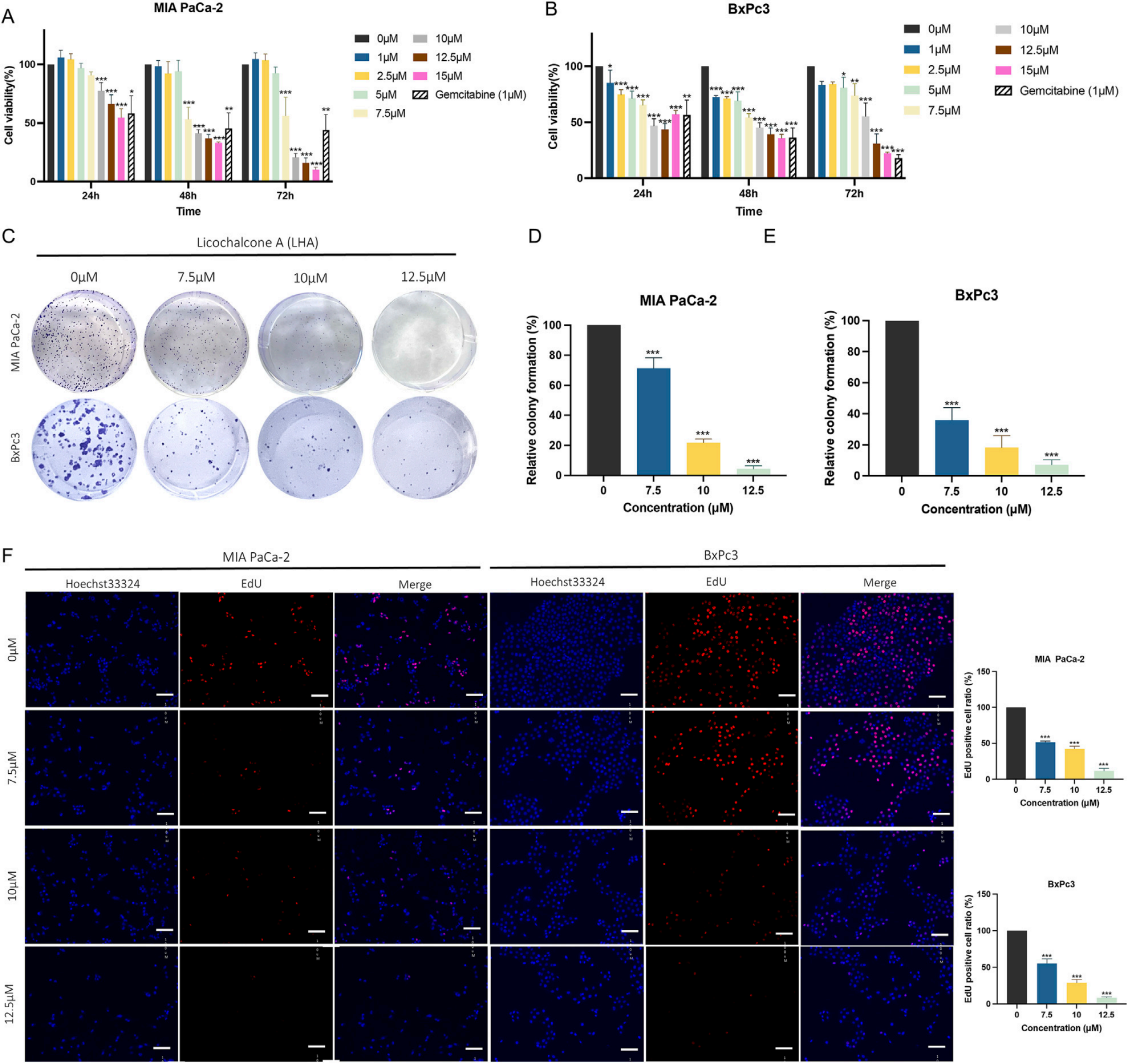
LHA inhibits the growth of pancreatic cancer cells. The cell viability of LHA on MIA PaCa-2 **(A)** and BxPc3 cells **(B)** was measured using MTT assay. Cell proliferation was determined using colony formation **(C–E)** and EdU assays **(F)**. The result in the treatment groups was expressed in percentage and compared to the corresponding controls. Data were expressed as mean ± S.D. of at least three independent experiments. *p < 0.05, **p < 0.01, and ***p < 0.001 vs. control.

The MTT assay was used to examine the cytotoxicity effect of LHA on two PDAC cell lines, MIA PaCa-2 and BxPc3. The results showed a clear dose-dependent cell inhibitory effect between the concentration of LHA (1 µM–15 µM) at 24, 48, and 72 h (Figures 1A,B). The IC_50_ of MIA PaCa-2 and BxPc3 at 48 h were 9.494 µM and 8.138 µM, respectively. These findings underscore the potential of LHA as a cytotoxic agent in PDAC treatment, leading us to select the concentration of LHA (7.5 µM, 10 µM, and 12.5 µM) for the subsequent experiments. Furthermore, the tumor colony formation and EdU assays were conducted to examine the effect of LHA on the proliferative ability of PDAC cells. These results showed a significant decrease in PDAC tumor colony formation in a dose-related manner. Specifically, BxPc3 cells demonstrated a more pronounced reduction in tumor colonies at the same 7.5 μM LHA concentration compared to MIA PaCa-2 cells. However, the reduction in tumor colonies was similar in both PDAC cell lines at the 10 μM and 12.5 μM LHA concentrations (Figures 1C–E). In the EdU proliferation assay, LHA reduced the EdU-positive MIA PaCa-2 cell ratio in a dose-related relationship. A concentration of 7.5 μM LHA significantly reduced the EdU-positive cell ratio in MIA PaCa-2 cells by approximately 50%, with comparable results observed in the BxPc3 cell line (Figure 1F). Taken together, LHA inhibited the growth of pancreatic cancer cells.

### 3.2 LHA induced protective autophagy in PDAC cells

**FIGURE 2:**
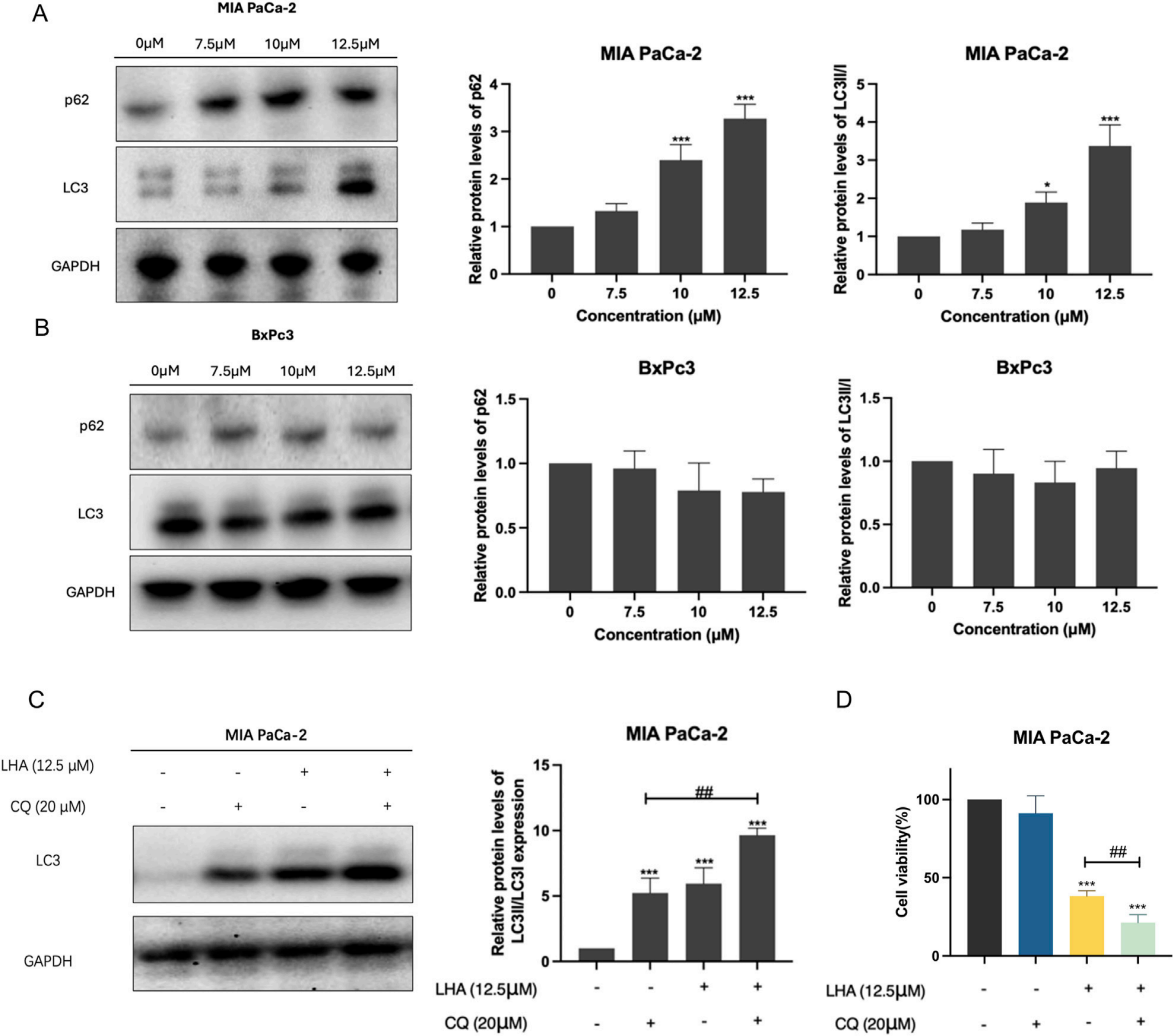
LHA induced protective autophagy in PDAC cells. **(A,B)** MIA PaCa-2 and BxPc3 cells were treated with LHA (0, 7.5, 10, and 12.5 μM) for 24 h, and the autophagy-associated protein markers of p62 and LC3II were tested using Western blotting. **(C)** The protein expression of LC3II in MIA PaCa-2 cell was measured after co-treatment with LHA and CQ (20 μM) using Western blotting. **(D)** The MIA PaCa-2 cell viability was measured after co-treatment with LHA and CQ (20 μM) using MTT assay. Data were expressed as mean ± S.D. of at least three independent experiments. *p < 0.05, **p < 0.01, and ***p < 0.001 vs. control. ^*#*^ *p* < 0.05, ^*##*^ *p* < 0.01, and ^*###*^ *p* < 0.001.

Autophagy is usually upregulated in PDAC cells (Piffoux et al., 2021). Hence, we will investigate the effect of LHA on autophagy in PDAC cells. Our results found that LHA significantly increased the expressions of autophagy-related proteins p62 and LC3II in a dose-dependent manner in MIA PaCa-2 cells; however, it did not show a noticeable alteration in BxPc3 cells (Figures 2A,B). Autophagy plays a dual role in cancer, involving tumor promotion and tumor suppression (Yun et al., 2021). To further explore the role of autophagy in MIA PaCa-2 cells, the autophagy inhibitor CQ was added to impair autophagy by inhibiting the fusion of autophagosomes and lysosomes. The results demonstrated that the co-treatment of CQ and LHA enhanced the protein expression of LC3II significantly compared with treatment with CQ alone (Figure 2C). These results suggested that LHA might induce autophagy flux in MIA PaCa-2 cells. From the MTT assay result, the cell viability of co-treatment with CQ and LHA was reduced by 15% compared to the LHA treatment alone (Figure 2D). These results implied that LHA-induced protective autophagy in the MIA PaCa-2 cells and the autophagy inhibitor could improve its cytotoxicity for PDAC.

### 3.3 LHA induced pyroptosis in PDAC cells

**FIGURE 3:**
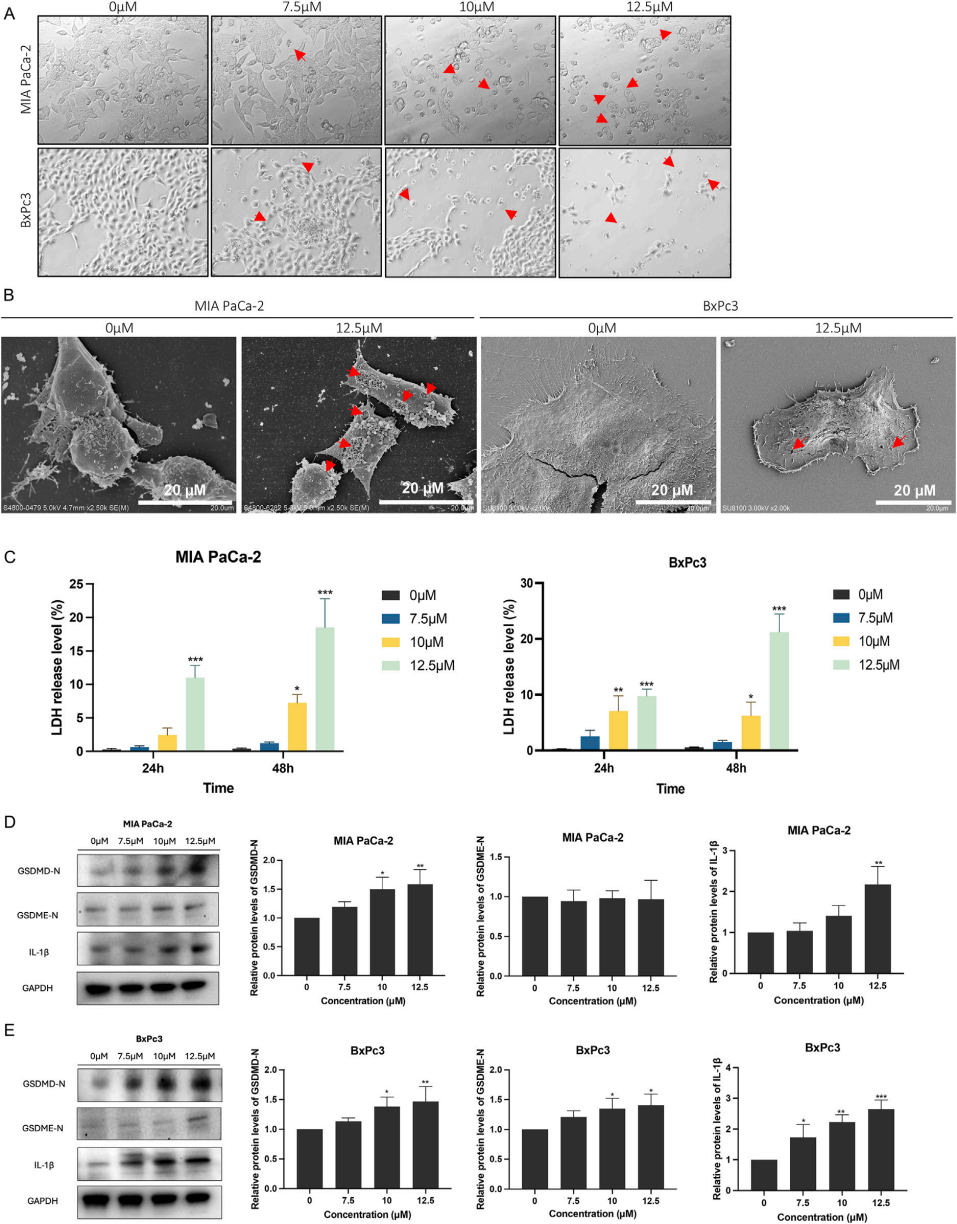
LHA induces pancreatic cancer cell pyroptosis. **(A)** MIA PaCa-2 and BxPc3 cells were treated with LHA (0, 7.5, 10, and 12.5 μM) for 48 h and captured under a light microscope. Red arrow: pyroptotic cell. **(B)** MIA PaCa-2 and BxPc3 cells were treated with LHA (0 and 12.5 μM) for 48 h and captured under an SEM. Red arrow: pyroptotic pores. **(C)** The percentage of LDH release was measured in MIA PaCa-2 and BxPc3 cell culture medium at 24 h and 48 h, respectively. **(D,E)** MIA PaCa-2 and BxPc3 cells were treated with LHA (0, 7.5, 10, and 12.5 μM) for 48 h. The pyroptosis-associated proteins of GSDMD-N, GSDME-N, and IL-1β were displayed using Western blotting. Data were expressed as mean ± S.D. of at least three independent experiments. *p < 0.05, **p < 0.01, and ***p < 0.001 vs. control.

Given that LHA can inhibit PDAC cell growth, further investigation was undertaken to ascertain the specific type of cell death mediated by LHA. Flow cytometry analysis of apoptosis demonstrated minimal changes in early and late apoptosis, suggesting that apoptosis is not the dominant mode of cell death induced by LHA (Supplementary Figure S1). After 48 h of LHA treatment, the pyroptosis cell morphology, involving cell swelling and large bubbles in the plasma membrane, could be observed under the light microscope in MIA PaCa-2 and BxPc3 cells, as shown. Furthermore, the number of cells with pyroptosis features increased with the increase in the concentration of LHA (Figure 3A). SEM analysis revealed the morphological features of LHA-induced pyroptosis in MIA PaCa-2 and BxPc3 cells (Figure 3B). Furthermore, the LDH release level was increased according to the concentration of LHA and time in both MIA PaCa-2 and BxPc3 cells (Figure 3C). Western immunoblotting was used to further determine the expressions of proteins involved in LHA-induced PDAC pyroptosis. Our data showed that LHA upregulates GSDMD-N and IL-1β expressions in MIA PaCa-2 cells (Figure 3D), as well as GSDMD-N, GSDME-N, and the downstream IL-1β expressions in BxPc3 cells (Figure 3E). However, these only significantly increased after the LHA treatment for 48 h. To sum up, LHA induced pyroptosis in PDAC cells.

### 3.4 LHA obstructed cell migration on PDAC cells

**FIGURE 4:**
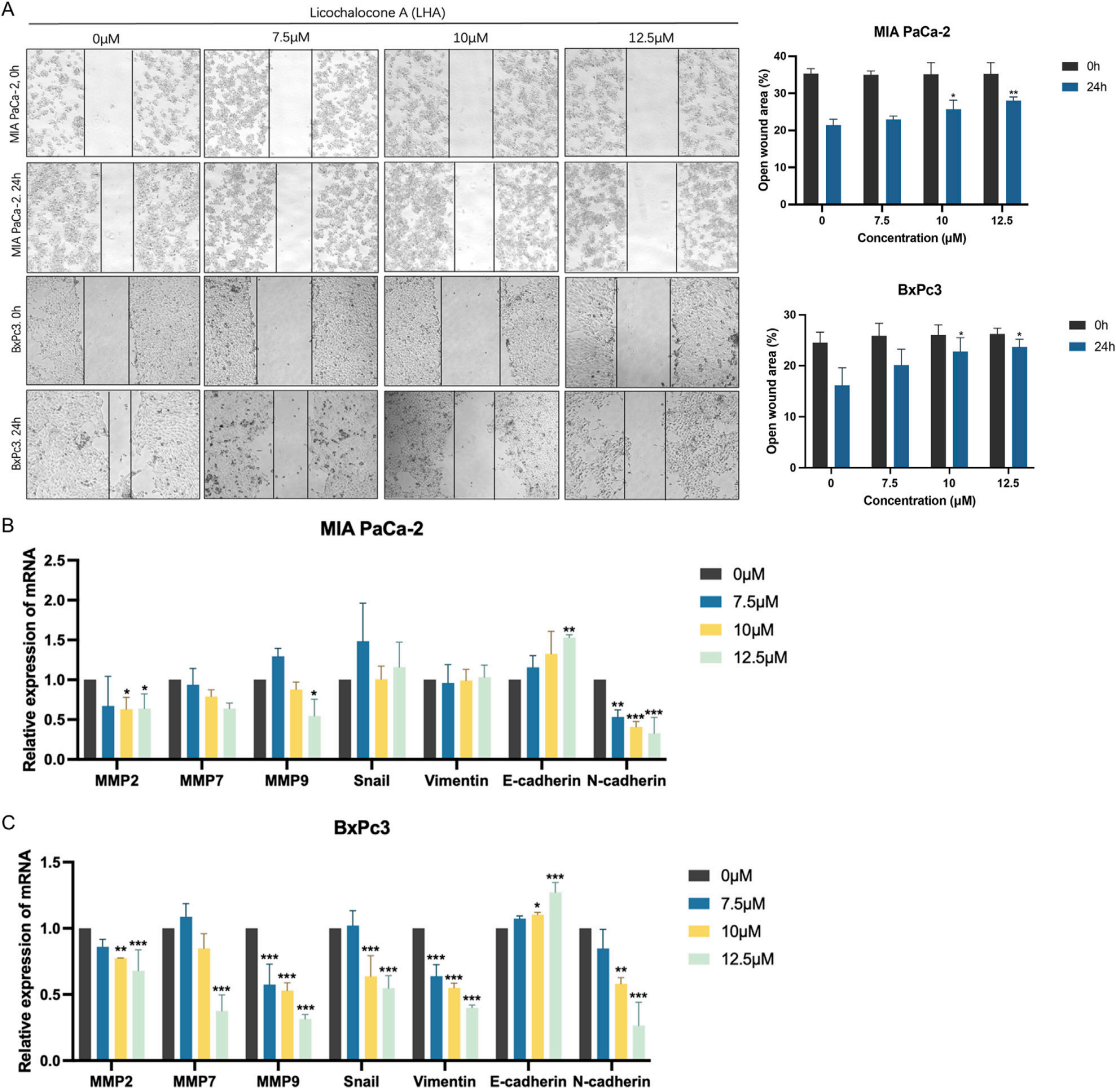
LHA suppresses the migration of pancreatic cancer cells. **(A)** The effect of LHA on the migration of pancreatic cancer cells was determined using wound-healing assay. Images were captured at two time points (0 and 24 h) after the LHA treatment. Representative photographs show the same area at time 0 to after 24-h incubation. **(B,C)** The genetic levels of cell migration markers, including MMP2, MMP7, MMP9, Snail, vimentin, E-cadherin, and N-cadherin, were decided using the RT-PCR. Data were expressed as mean ± S.D. of at least three independent experiments. *p < 0.05, **p < 0.01, and ***p < 0.001 vs. control.

In general, most PDAC patients are diagnosed with Stage IV disease, which has already metastasized to the surrounding organs, when they seek medical advice. Therefore, whether the drug can inhibit cancer cell metastasis and invasion is a crucial factor. The wound-healing migration assay was used to assess the ability of LHA to suppress cell migration in PDAC cells. The control group showed a more rapid closure of the open region (wound) after 24 h, but the group holding 12.5 µM LHA showed almost no migration in both MIA PaCa-2 and BxPc3 cell lines (Figure 4A). It revealed that LHA suppresses PDAC cell migration. Real-time PCR was used to further evaluate migration-related gene markers. Our data showed that LHA treatment significantly downregulated the expressions of matrix metalloproteinases (MMP2, MMP7, and MMP9) in both cell lines (Figures 4B,C). Furthermore, LHA induced notable changes in epithelial–mesenchymal transition (EMT) markers, including increased E-cadherin expression and decreased N-cadherin levels. These molecular alterations were consistent with the observed reduction in cell migration capacity. Notably, LHA downregulated the gene expressions of EMT-related factors such as Snail and vimentin of BxPc3 cell (Figures 4B,C). Together, these findings suggest that LHA effectively suppresses PDAC cell migration, indicating its potential as a therapeutic agent for controlling pancreatic cancer metastasis.

### 3.5 LHA reduced the expressions of eEF2K and p-eEF2 proteins in PDAC cells

**FIGURE 5:**
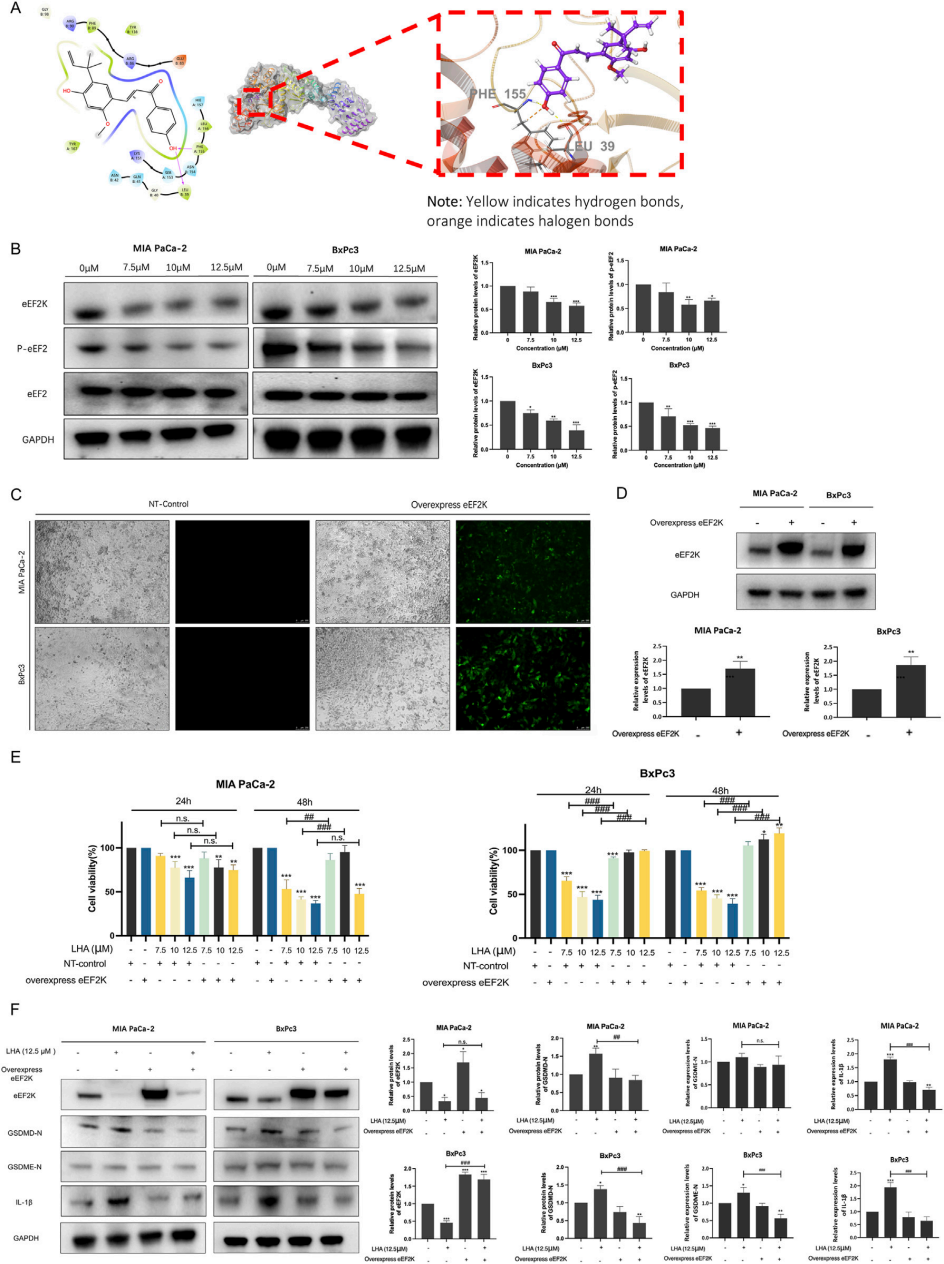
LHA induces pyroptosis through targeting eEF2K in PDAC cells. **(A)** Molecular docking of LHA on eEF2K protein. Predicted two-dimensional and three-dimensional crystal structures of LHA (PubChem CID: 5318998) and the eEF2K (PDB ID: 7SHQ) complex. **(B)** MIA PaCa-2 and BxPc3 cell lines were treated with LHA (0, 7.5, 10, and 12.5 μM) for 48 h. The expressions of eEF2K, p-eEF2, and eEF2 were examined using Western blotting. Data were expressed as mean ± S.D. of at least three independent experiments. *p < 0.05, **p < 0.01, and ***p < 0.001 vs. control. **(C)** MIA PaCa-2 and BxPc3 cell lines were transfected using lentivirus vectors with the overexpressed human eEF2K gene, and the images were captured using the fluorescent microscope after 72 h of transfection. **(D)** MIA PaCa-2 and BxPc3 with or without eEF2K overexpression. The expressions of eEF2K were examined using Western blotting. Data were expressed as mean ± S.D. of at least three independent experiments. **p < 0.01 vs. control. **(E)** The cell viability of MIA PaCa-2 and BxPc3 cell lines with or without overexpression of eEF2K was examined using MTT assay. Data were expressed as mean ± S.D. of at least three independent experiments. *p < 0.05, **p < 0.01, and ***p < 0.001 vs. control. ^*#*^ *p* < 0.05, ^*##*^ *p* < 0.01, and ^*###*^ *p* < 0.001 vs. overexpression eEF2K with the same LHA concentration. **(F)** MIA PaCa-2 and BxPc3 with or without eEF2K overexpression with LHA (0 and 12.5 μM) for 48 h. The expressions of eEF2K, GSDMD-N, GSDME-N, and IL-1β were examined using Western blotting. Data were expressed as mean ± S.D. of at least three independent experiments. *p < 0.05, **p < 0.01, and ***p < 0.001 vs. control. ^*##*^ *p* < 0.01 and ^*###*^ *p* < 0.001 vs. overexpression eEF2K with the same LHA concentration.

To explore the molecular mechanism of LHA’s anti-pancreatic cancer effect, molecular docking analysis was performed. The results demonstrated that LHA stably binds to the active pocket of eEF2K, forming hydrophobic interactions with residues PHE89, LEU156, and PHE155. Additionally, LHA established one hydrogen bond with LEU39 and a hydrogen bond and a halogen bond with PHE155 (Figure 5A). The XP docking score (−5.484 kcal/mol) and MM-GBSA binding free energy (−51.24 kcal/mol) further support the stability of this interaction. According to established criteria, an XP Gscore < −6 kcal/mol indicates stable ligand–protein binding, while an MM-GBSA dG Bind < −30 kcal/mol confirms low binding free energy and robust complex stability. Collectively, these data validate the strong and stable binding of LHA to eEF2K. From the earlier research study, it was proven that eEF2K was overexpressed in pancreatic cancer, and it was a crucial marker related to the low survival rate. Meanwhile, we demonstrated that LHA could stably bind on eEF2K by molecular docking. The result showed that LHA downregulated eEF2K and p-eEF2 protein expression in a concentration-dependent manner in PDAC cell lines (Figure 5B). These data implied that LHA downregulated eEF2K expression and induced translation elongation in PDAC.

To further validate the role of eEF2K in LHA-mediated anti-tumor effects, we established eEF2K-overexpressing PDAC cell lines using lentiviral vectors. A wide range of eEF2K signals were captured in transfected cells after 72 h, while no detectable signals were observed in the control group (Figure 5C). Western blot analysis further confirmed the successful generation of eEF2K-overexpressing PDAC cell lines (Figure 5D). Notably, eEF2K overexpression significantly attenuated the cytotoxic effects of LHA in both PDAC cell lines. In MIA PaCa-2 cells, although no significant differences were observed at 24 h, eEF2K overexpression markedly increased the cell viability at 48 h post-LHA treatment. In particular, compared to LHA-treated control cells, the eEF2K-overexpressing cells demonstrated increased viability by 33%, 54%, and 11% when treated with 7.5 μM, 10 μM, and 12.5 μM LHA, respectively (Figure 5E). The reversal effect was even more pronounced in BxPc3 cells. Although minimal changes were observed at 24 h, eEF2K overexpression not only counteracted the cytotoxicity of LHA but also promoted cell proliferation at 48 h, with increased viability of 5.6%, 12.3%, and 19.3% at 7.5 μM, 10 μM, and 12.5 μM LHA concentrations, respectively, compared to controls (Figure 5E). To conclude, eEF2K overexpression significantly reverses the cytotoxicity of LHA in PDAC cells.

To elucidate the mechanistic relationship between eEF2K and pyroptosis in LHA-mediated cell death, we examined the expressions of pyroptosis markers including GSDMD-N, GSDME-N, and cleaved IL-1β using Western blot analysis. In control PDAC cells, treatment with 12.5 μM LHA significantly upregulated the expression of GSDMD-N and cleaved IL-1β both in MIA PaCa-2 and BxPc3 cells, as well as GSDME-N in the BxPc3 cells, indicating the activation of pyroptotic cell death (Figure 5F). However, this effect was markedly attenuated in eEF2K-overexpressing cells, where LHA treatment failed to induce significant changes in those protein levels compared to untreated controls (Figure 5F). These findings provide compelling evidence that LHA-induced pyroptosis in PDAC cells is mechanistically linked to eEF2K inhibition, suggesting that eEF2K serves as a critical regulatory node in the pyroptotic cell death pathway activated by LHA.

### 3.6 LHA inhibited tumor growth in PDAC xenograft models

**FIGURE 6:**
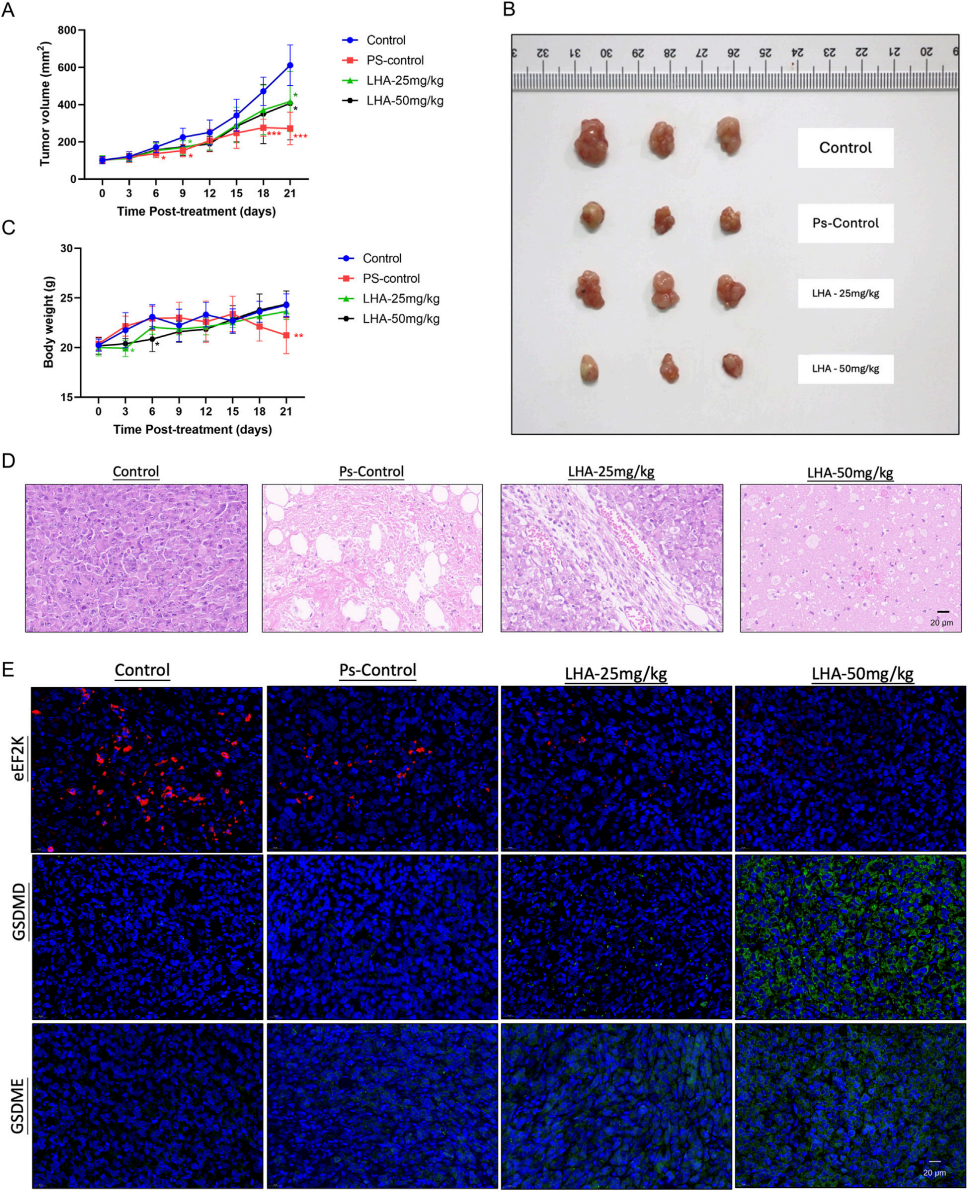
Inhibitory effect of LHA on tumor growth in the PDAC xenograft model. **(A)** Tumor volume, Ps-control (gemcitabine: 15 mg/kg) **(B)**, tumor photograph, and **(C)** body weights of four groups of mice are displayed (n = 6). **(D)** Tissues from the xenograft mouse model were stained with hematoxylin and eosin. After the stain, the slides were observed and captured under the microscope with ×63 magnification. **(E)** Tissues from the xenograft mouse model were stained with eEF2K (red spot), GSDMD, and GSDME (green spot). The nucleus was stained with DAPI and shown as blue fluorescence. Three random images were captured under the fluorescence microscope with ×63 magnification. Data were expressed as mean ± S.D, *p < 0.05, **p < 0.01, and ***p < 0.001 vs. control.

To evaluate the therapeutic efficacy of LHA *in vivo*, we established xenograft models using Balb/c nude mice. Gemcitabine (15 mg/kg), the current first-line chemotherapeutic agent for PDAC, served as a positive control. By day 21 post-treatment, both the LHA-treated groups and the gemcitabine group demonstrated a marked reduction in tumor volume (Figures 6A,B). Both treatments of low-dose (25 mg/kg) and high-dose (50 mg/kg) LHA significantly reduced tumor mass compared to the control group (Figures 6A,B; Supplementary Figure S2). Assessment of body weight trajectories revealed that, unlike gemcitabine which induced significant weight loss after day 21, LHA treatment did not cause substantial body weight reduction throughout the experimental period (Figure 6C), suggesting that LHA may exhibit a more favorable preliminary toxicity profile than gemcitabine. However, further studies incorporating extended observation windows and multi-organ toxicity assessments are warranted to fully characterize its safety.

The internal structure of the tumor, cell distribution, and cell shape could become easily visible after H&E staining. In the control group, the cell density was the most concentrated. However, the distribution of cells in the LHA-50 mg/kg group was more scattered. The entire construction of the PDAC cell could not be observed, and the size of the cell nuclei shrunk significantly (Figure 6D). These results suggested that LHA could change the internal tumor structure and inhibit tumor proliferation and development.

Next, we harvested tumor tissues and stained them with eEF2K, GSDMD, and GSDME immunofluorescence markers. After the drug treatment, whether it was gemcitabine or LHA, the expression level of eEF2K was reduced compared to the PDAC control group. However, the LHA showed a more potent eEF2K inhibition than that in the positive control group (Figure 6E). In addition, the GSDMD and GSDME expressions in the control and positive control groups are similar; these results suggested that gemcitabine might not induce PDAC death through pyroptosis *in vivo*. In the LHA situation, the GSDMD and GSDME expressions were enhanced compared to those of the control group, especially in the high-dosage group (Figure 6E). This observation provides compelling evidence that LHA induces pyroptosis in PDAC cells *in vivo*, whereas the anti-tumor effects of gemcitabine appear to be independent of pyroptotic cell death. These findings highlight a novel mechanism of action for LHA in PDAC treatment, potentially opening new therapeutic avenues for this aggressive malignancy.

## 4 Discussion

Late diagnosis and a high recurrence rate lead to a 5-year survival rate of pancreatic cancer, only approximately 11% (Siegel et al., 2023). The current treatment approaches for pancreatic cancer include resection surgery and chemotherapy drugs. However, their effectiveness must be improved to significantly extend the life span of patients. Thus, recent research is devoted to discovering some drugs with high potency and mild side effects for pancreatic cancer. In this study, we present compelling evidence of the anti-pancreatic cancer potential of LHA *in vitro* and *in vivo*, offering a ray of hope for improved patient outcomes.

In the earlier part of our study, we proved that LHA had an inhibition effect on PDAC cell viability by using an MTT assay. Meanwhile, the relative PDAC cell colony formation and EdU positive-cell ratio reduced significantly after the LHA treatment. These data implied that LHA was cytotoxic to PDAC cells and suppressed PDAC cell proliferation to achieve anticancer effects. Autophagy has a dual role in tumors, involving tumor protection and suppression, and it has already been claimed that autophagy is promoted in PDAC (Piffoux et al., 2021). Our findings align with this duality: LHA induced a dose-dependent upregulation of autophagy-related proteins p62 and LC3II in MIA PaCa-2 cells, but not in BxPc3 cells (Figures 2A,B). This cell line-specific autophagy activation likely explains the differential cytotoxic sensitivity observed in IC_50_ values (9.494 µM for MIA PaCa-2 vs. 8.138 µM for BxPc3 at 48 h). The higher IC_50_ value in MIA PaCa-2 suggests that LHA-triggered protective autophagy partially mitigates its cytotoxicity in this cell line, whereas the absence of autophagy induction in BxPc3 cells allows LHA to exert stronger cytotoxic effects through alternative pathways, such as pyroptosis. This hypothesis is further supported by the enhanced cytotoxicity observed when combining LHA with the inhibitor CQ in MIA PaCa-2 cells: co-treatment synergistically increased LC3II accumulation (indicative of blocked autophagic flux) and reduced cell viability by 15% compared to treatment with LHA alone (Figures 2C,D), confirming that autophagy serves as a survival mechanism in MIA PaCa-2 cells. Notably, pyroptosis was more pronounced in BxPc3 cells than in MIA PaCa-2 cells. This is evidenced by the broader activation of pyroptotic markers in BxPc3 cells, including GSDMD-N, GSDME-N, and higher IL-1β levels. In contrast, MIA PaCa-2 cells exhibited only GSDMD-N elevation. The enhanced pyroptotic response in BxPc3 cells correlates with its lower IC_50_ value and the absence of autophagy induction, suggesting that in BxPc3 cells, the cytotoxicity of LHA is predominantly driven by pyroptosis rather than autophagy modulation.

GSDMD and GSDME are the representative markers of pyroptosis. Once pyroptosis is promoted, these markers are cleaved into the C-terminus and N-terminus (Broz et al., 2020; Liu et al., 2024). After that, the N-terminus stimulates the pore formation on the cell to release cytokines, such as IL-1β, to create an inflammation environment (Fang et al., 2020). This study proved LHA-induced pyroptosis in PDAC by observing the change in cell morphology and pyroptosis-related protein expression. Some dominant pyroptosis morphologies, for example, cell swelling and pore formation, could be captured in PDAC cells using the light microscope and SEM. In addition, the protein expression levels of GSDMD or GSDME N-terminus and IL-1β were enhanced after the treatment of PDAC cells with LHA. In conclusion, pyroptosis was triggered in PDAC by LHA. Nevertheless, further finding the upstream regulator that induces pyroptosis still requires time.

Instead of the rapid growth and development of PDAC cells, invasion and migration are also notable factors that lead to a low survival rate of PDAC. In our study, the mRNA expressions of MMP2, MMP7, and MMP9 were decreased by LHA to suppress the PDAC migrations, and it was consistent to the earlier report, which pointed out that cellular migration was reduced when MMP2 and MMP expressions were inhibited (Webb et al., 2017). A “cadherin-switch” is critical to cell migration and invasion. In a progression of tumor migration, the expression of E-cadherin will decrease, and that of N-cadherin will increase. Thus, the tumor could migrate to neighboring organs or tissues through the blood vessel without the recognition from immune cells such as natural killer (NK) cells and T cells (Eksi et al., 2025; Na et al., 2020; Yu W. et al., 2019). We found that LHA upregulated the mRNA expression level of E-cadherin and downregulated that of N-cadherin to repress the PDAC migration. In conclusion, LHA inhibits PDAC cell migration through regulated migration-related markers.

From the recent research, overexpression of eEF2K was demonstrated in pancreatic cancer (Wang N. et al., 2024). It is a crucial regulator of cell autophagy, apoptosis, and cell cycle and a reason for causing a low survival rate (Eksi et al., 2025; Temme and Asquith, 2021; Jia et al., 2024). In our study, molecular docking predicted a stable bonding between LHA and the active pocket surface of the eEF2K protein. The protein expressions of eEF2K and p-eEF2 decreased in PDAC cells according to the concentration of LHA. Furthermore, an overexpression of eEF2K was constructed in PDAC cells using lentivirus. Compared to the PDAC cells without eEF2K overexpression, the cell viability with the exact LHA dosage was increased in eEF2K-overexpression cell lines. This result proved that the overexpression of eEF2K enhanced PDAC cell viability. Finally, we confirmed that eEF2K was a mediator of LHA pyroptosis. At the 12.5 μM concentration of LHA, the pyroptosis-related protein markers only increased in the PDAC cells with normal eEF2K expression, not the overexpression of eEF2K cell lines. The expression of eEF2K is usually upregulated to inhibit the translation elongation in nutrient limitation conditions in cancer for saving energy to enhance the cancer survival rate. However, this result implied that LHA-induced PDAC cell death was caused by energy and nutrient uptake within the cancer cells to promote translation elongation.

In the PDAC xenograft model, Balb/c mice were used to prove the effectiveness of LHA *in vivo*. The mice treated with low dosage (25 mg/kg) or high dosage (50 mg/kg) of LHA showed a significant reduction in tumor mass compared to the control group. This result implied that LHA has a strong anti-PDAC effect, consistent with the *in vitro* test. We discovered accidentally that gemcitabine caused an apparent weight loss in mice after the post-treatment day 21. This outcome might relate to the side effects of gemcitabine. LHA treatment did not induce any change in body weight in mice. Moreover, the internal tumor structure of mice loosens with administration of either gemcitabine or LHA compared with the control. LHA also decreased the expression of eEF2K and increased the expression of pyroptosis markers in the tumor tissues from the PDAC xenograft models.

## 5 Conclusion

In conclusion, our study demonstrates that LHA exerts potent anti-PDAC activities by suppressing cell proliferation, inducing pyroptosis and repressing cell migration, with eEF2K serving as a critical molecular target. These findings not only advance our understanding of the role of eEF2K in pyroptotic cell death but also establish LHA as a promising natural eEF2K inhibitor. The superior efficacy and favorable safety profile observed both *in vitro* and *in vivo* suggest that LHA warrants further investigation as a potential therapeutic agent for PDAC treatment. This study thus provides a strong foundation for future clinical development of LHA-based therapeutic strategies in pancreatic cancer.

## Data Availability

The original contributions presented in the study are included in the article/Supplementary Material; further inquiries can be directed to the corresponding author.

## Abbreviations

AKT, protein kinase B; ANOVA, analysis of variance; BSA, bovine serum albumin; CQ, chloroquine disphosphate salt; EDTA, tthylenediamine tetraacetic acid; EdU, 5-ethynyl-2′-deoxyuridine; eEF2, eukaryotic elongation factor-2; eEF2K, eukaryotic elongation factor-2 kinase; EMT, epithelial–mesenchymal transition; GSDMD, gasdermin D; GSDME, gasdermin E; IL, interleukin; LC3, microtubule-associated protein 1A/1B-light chain 3; LDH, lactate dehydrogenase; LHA, licochalcone A; MMP, matrix metalloproteinases; mTOR, mammalian target of rapamycin; mTORC1, mammalian target of rapamycin complex 1; Ni, nickel; NK, natural killer; PDAC, pancreatic ductal adenocarcinoma; PFA, paraformaldehyde; PI3, phosphoinositide 3; PI3K, phosphoinositide 3-kinase; PSN, penicillin–streptomycin–neomycin; RPMI, Roswell Park Memorial Institute; SEM, scanning electron microscopy.
