# Supplementary material for: Licochalcone A suppresses pancreatic ductal adenocarcinoma progression by targeting eEF2K-mediated pyroptosis
Source: Front Pharmacol. 2025 Jun 11;16:1595686. doi: 10.3389/fphar.2025.1595686 (PMC12187834; doi:10.3389/fphar.2025.1595686)
Supplement: Supplementary file 1 [file Supplementaryfile1.docx]

Fig.S1. Apoptotic ratio was measured using flow cytometer after LHA treatment for 24 h. Data were expressed as mean ± S.D. of at least three independent experiments. No significant difference was found between LHA treatment and Control (0 µM).





Fig.S2. Tumor image and tumor weight (n=6 mice/group). Data are presented as mean ± SD, **p* < 0.05, ****p*<0.001 vs. control.
